# Tracking Down Nonresponsive Cortical Neurons in Cochlear Implant Stimulation

**DOI:** 10.1523/ENEURO.0095-17.2017

**Published:** 2017-06-27

**Authors:** Charlotte Amalie Navntoft

**Affiliations:** Department of Biomedicine, Basel University, Basel, 4056, Switzerland

**Keywords:** Auditory cortex, cochlear implants, interneurons, marmoset, sideband inhibition

## Abstract

**Figure:**
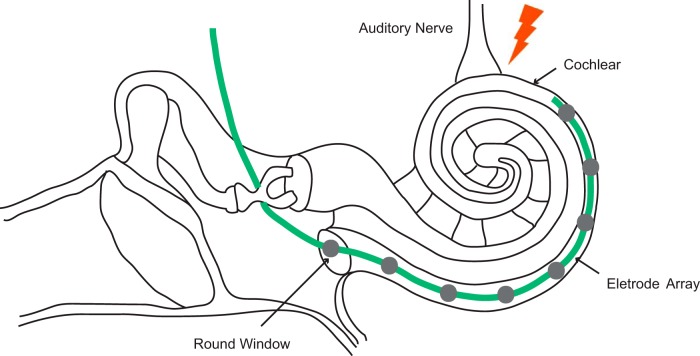

## Significance Statement

The cochlear implant (CI) can restore a sense of hearing to deaf people. Although it works well in quiet situations, most CI users still struggle to follow conversations in noisy environments and perceive music. One challenge in CI stimulation is that the brain must resolve and interpret the signal to generate meaningful and goal-oriented hearing. How efficient this process is and how the underlying neuronal circuits respond, or fail to respond, to such input compared with normally transmitted sound is essentially unknown. Understanding these central auditory mechanisms is crucial for the further development of CIs. In a recent study published in *The Journal of Neuroscience*, Johnson et al. (2016) investigated how CI stimulation engages the auditory cortex in awake marmosets. There were two main findings: first, CIs are surprisingly inefficient in activating cortical neurons that normally respond to sound; second, CI-responsive neurons are functionally different from non–CI-responsive neurons. Here, we discuss the results and hypothesize how inhibition could be involved the brain’s response to CIs. 

## 

Sound travels from its source to the ear, and further on eventually to the brain, through a chain of biological mechanisms that convert vibrations in the air into nerve impulses. In deaf people, the link between sensory hair cells and the auditory nerve in the cochlear is often broken (or the terminals of the auditory nerve are simply gone), and the auditory input therefore never reaches the brain. Cochlear implants (CIs) bypass this missing link by directly stimulating the auditory nerve. To date, this approach has restored hearing sensation to >300,000 severely and profoundly deaf people.

Present-day design of CIs is based on a bottom-up approach in which the goal is to reproduce normal patterns of neural activity at the auditory periphery. In a recent study published in *The Journal of Neuroscience*, Johnson et al. (2016) used another way of thinking about CI design that includes mechanisms from the central auditory system, and not only its periphery. In such a top-down approach, one asks what input the central auditory system needs to perform optimally in a given situation (Wilson et al., 2011).

The common marmoset (*Callithrix jacchus*) is a valuable model of how the human brain processes auditory information: it is a highly vocal, communicative, and social nonhuman primate, and its hearing range and organization of the auditory cortex (AC) are similar to those of humans (de la Mothe et al., 2006; Osmanski and Wang, 2011). Furthermore, the use marmosets makes possible the determination of single-neuron responsiveness, which is extremely difficult or impossible to obtain in humans.

**Figure 1.**
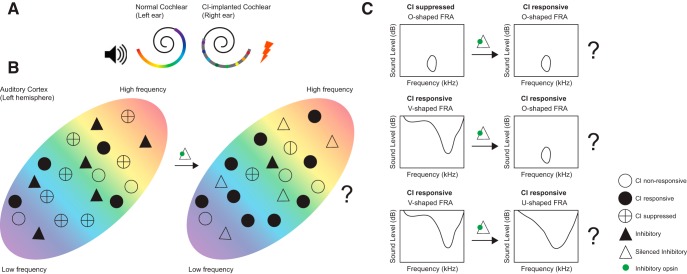
***A***, The setup used in Johnson et al., 2016. A CI implant with eight electrodes is implanted in the right ear, and the left ear is left acoustically intact. The perception of different frequencies is elicited by stimulating different electrodes along the tonotopic axis in the cochlea (color gradient). This strategy allows Johnson et al. to measure the response of single neurons in the left auditory cortex to both acoustic and CI stimulation in an awake marmoset, and thereby to examine the characteristics of neurons that respond, or fail to respond, to CI stimulation. Acoustic and CI stimuli were matched whenever possible. For CI stimulation, the response of a neuron is tested across different electrode positions and at multiple current levels, and analogously for acoustic stimulation, across a range of frequencies and at multiple sound levels. The CI electrode/frequency producing the significantly largest firing rate response is defined as the best electrode or best frequency of the neuron, for CI and acoustic stimulation, respectively. The receptive field of a neuron is described by electrode/frequency tuning curves across all current/sound levels. ***B***, Hypothesis: interneurons, particularly PV interneurons, are important for effective cortical response to CI stimulation. Left, CI stimulation is surprisingly inefficient in activating A1 neurons (black circles) because many neurons are suppressed (crossed circle) by inhibitory interneurons (filled triangles). Right, decreasing inhibitory GABAergic interneuron activity using either optogenetics or pharmacology will increase the effectiveness of CI stimulation, and in particular, likely yield more O-shaped neuron activity either from previously suppressed (top) or evolved from V-shaped neurons (middle; see text). However, decreased GABAergic inhibition would likely come at the cost of broader V-shape tuning in already CI-responsive cells (bottom). Filled circle, CI responsive neuron; open circle, CI nonresponsive neuron; crossed circle, suppressed neuron; filled triangle, active inhibitory neuron; open triangle, inactivated inhibitory cell; green circle, inhibitory opsin or pharmacological blockage.

Johnson et al. used a novel unilateral CI model, implanting an eight-electrode array in one ear of the marmoset and leaving the other ear intact. Taking advantage of the binaural properties of AC—neurons in one hemisphere are excited by inputs from both ears—the authors precisely located the primary auditory cortex (A1) and characterized the acoustic features and selectivity of A1 neurons to CI stimulation (Fig. 1*A*, *B*).

First, the authors investigated how efficient a CI is in transmitting information to the AC compared with acoustic stimuli. They recorded 1408 single neurons from the AC and classified them as either acoustic-driven (the neuron responded to tones or bandpass noise) or CI-driven (the neuron responded to electric stimulation of the implant). It is important to highlight that acoustic and CI stimulation are two different quantities (Wilson and Dorman, 2009), and the authors therefore carefully optimized stimuli parameters to match them as well as possible. One striking finding of the work is that CI stimulation was surprisingly inefficient at activating as many neurons as comparable acoustic stimulation. Why are some neurons, and not others, activated by a CI?

One possible explanation is related to the differences between contralateral and ipsilateral stimulation, with contralateral stimulation typically being much more effective and precise (Lui et al., 2015). Because their novel unilateral model allowed for testing each neuron’s response to both acoustic and CI stimulation, Johnson et al. could compare acoustic properties of nonresponsive and CI-driven cells. The assumed binaurality is a limitation of the paper. It may have been difficult to “functionally localize” responsive neurons, via acoustic stimulation, in the hemisphere contralateral to the implant because the response to ipsilateral acoustic stimulation is different. To address this issue, the authors could have recorded the cortical response first to contralateral acoustic stimulation and then to contralateral CI stimulation after CI insertion in the same ear.

A second possible explanation is that the CI has been activated only with a standard stimulation protocol and not a test battery of additional CI pulse variables (e.g., different pulse wave form shapes, pulse durations, pulse stimulation rates, amplitude modulation), which potentially could engage neurons in a more similar way (higher number of activated neurons, more focused response in the tonotopic map, higher firing rate, etc.). A third possibility is lack of cortical adaptation to CI stimulation, discussed below.

The response of a neuron to pure tones of varying frequency and level, known as its frequency response area (FRA), is a fundamental receptive field measure in the auditory system (Sutter et al., 1999). Neurons with so-called V-shaped FRAs respond to more frequencies with increasing sound levels, whereas O-shaped FRAs are narrowly tuned to sound frequency and level (Sadagopan and Wang, 2008). That CI stimulation mainly engaged neurons with V-shaped FRAs while nonresponsive cells typically had O-shaped FRAs supports the authors’ argument that CI stimulation differs in some fundamental ways from the stimulation that results from actual sounds (Johnson et al., 2016).

Why do neurons with small and selective receptive fields not respond to CI stimulation? Are they simply not activated, or are they suppressed? It is well established that the interaction between inhibition and excitation plays a crucial role in shaping and refining the brain’s representation of sensory stimulus attributes (Wehr and Zador, 2005). Nonresponsive cells were mainly located in upper cortical layers and CI-responsive cells in middle thalamo-recipient layers, suggesting that the CI signal is received by cortical structures but does not progress to further intracortical processing. In A1, so-called sideband inhibition helps to sharpen the tuning of local neuronal responses and appears as suppression of A1 neurons by off-frequency components (Sutter et al., 1999; Wang et al., 2000; de la Rocha et al., 2008). A big issue with CI stimulation is that the applied current spreads over a wide area, stimulating a large neuronal population (Landsberger et al., 2012), so the authors proposed that this spread activates an off-frequency component suppressive to neurons. Using broadband acoustic noise to mimic the broad CI stimulation and a two-tone suppression protocol to probe sideband inhibition, they found that CI nonresponsive neurons were also suppressed by broadband stimuli and more easily in the two-tone protocol than in CI-only driven cells. Taken together, the results suggest that the widespread current after CI stimulation activates neighboring off-frequency components that eventually suppress neuronal response to the incoming signal.

These results provide valuable insight into how CI only partially engages the brain at the level of single neurons. Below, I place the findings into perspective and propose future experiments.

The finding that CI-mediated inhibition overwhelms excitation leaves many questions unanswered. Is this misbalance related to the strength of—or the timing between—excitation and inhibition? Which cell types and circuits are involved? Will less inhibition improve efficiency and selectivity of CI stimulation, and eventually improve perception of CI input?

To address the first question, one could look at the temporal dynamics of O- and V-unit population responses on CI stimulation as in Sadagopan and Wang (2010). That study examined how FRA shape changes over the duration of the response to played sound using population-average FRAs in small time bins. In addition to the well-documented short-latency sideband suppression, they uncovered long-latency suppressions caused by single-tone stimulation. Interestingly, such long-latency suppressions also included monotonically increasing suppression with sound level both on–best frequency (BF) and off-BF. As the CI mainly activated V-unit neurons with monotonic acoustic rate-level functions, this suppressive effect could contribute to the lack of CI efficiency. Sadagopan and Wang (2010) also observed that over time a V-unit response evolved to an O-unit-like response. Further analysis of the present data could therefore investigate whether CI stimulation might fail to promote this process.

In the AC, synaptic inhibition is known to be involved in shaping receptive fields, controlling gain, and promoting temporal precision (Moore and Wehr, 2013; Aizenberg et al., 2015). The overall inhibitory tone is orchestrated by locally acting GABAergic interneurons. The most common types are parvalbumin-positive interneurons (PVs) that target the pyramidal cell bodies and gate feedforward thalamocortical auditory output of layer III/V neurons (Hamilton et al., 2013). In particular, PV activity is thought to be crucial for intensity tuning and to contribute to sideband inhibition and response timing (Moore and Wehr, 2013; Aizenberg et al., 2015). We therefore hypothesize that PV interneurons might play key roles in the observed inefficient cortical response to CIs. This hypothesis can be explored optogenetically via fast and efficient control of targeted cell types and, importantly, the opportunity to probe causal relations between various actors and actions of interest. A chronic optogenetic model has recently been developed in marmosets (MacDougall et al., 2016), and viral targeting of interneurons in marmosets is in its early phases (Dimidschstein et al., 2016). In the near future, it should be possible to optogenetically manipulate PV activity in AC while recording in AC of a CI-implanted animal (Fig. 1). If PV interneurons contribute to the lack of CI response, inhibiting PV interneurons should increase the effectiveness of CI stimulation and, in particular, might yield more O-unit activity either previously suppressed or evolved from V-shaped neurons. Until such an experiment is feasible, blocking all interneurons pharmacologically (GABAergic inhibitor) on CI stimulation in awake marmosets could be an interesting first step in understanding the role of interneurons (not just PV) in A1. Nonetheless, decreased GABAergic inhibition would likely come at the cost of broader V-shape tuning in already CI-responsive cells, as previously reported (Wang et al., 2000). Indeed, a finely tuned balance between inhibition and excitation is needed to produce an optimal cortical response after CI stimulation.

Hearing with a CI takes time and practice, because the auditory system must adapt to reinterpret the new auditory input as meaningful sound (Fallon et al., 2008; Kral and Sharma, 2012). Therefore, an important caveat of the work by Johnson et al. (2016) is that although recordings were made up to 14 mo after CI implantation, animals received only passive stimulation during the experimental sessions and not during engagement in a task, communication with conspecifics, or any other interaction with the surrounding environment. The results might thus relate to mechanisms in a recently implanted CI user, before any experience and behavioral-dependent plasticity takes place, rather than in an experienced CI user whose brain has adapted to make sense of CI stimulation.

The authors found that neurons sharply tuned to frequency and sound level (O-shaped FRAs) are poorly activated by CI stimulation, but whether this is a result of (1) cortical CI-evoked response or (2) lack of plastic changes is difficult to tell. In favor of the first statement, many CI users face challenges hearing in dynamic and noisy environments, which is partially due to limited dynamic range (5–15 dB compared with 0–120 dB in normal hearing) and a poor ability to discriminate steps in amplitude of a sound (Moore, 2003; Wilson et al., 2011). As these phenomena require fine and robust representation of sound level, it is likely that a general lack of O-unit response could underlie these perceptual limitations.

On the other hand, supporting the second statement, Polley et al. (2006) have shown that behavioral training in rats caused overrepresentation of the target-specific sound level in the AC (Polley et al., 2006), and plasticity is known to reorganize receptive fields in the AC (Froemke, 2015). Taken together, these data suggest that the marmoset AC in the study by Johnson et al. (2016) has a history of experience with real sounds but not the kind of stimulation produced by CIs. Future experiments using the CI marmoset model could test the hypothesis that O-units are important for CI performance in noisy surroundings. This approach might also reveal whether general experience and behavioral testing with chronic CI stimulation can engage more O-units, and if so, how this would impact perception of CI stimulation. Finally, using the top-down approach described by Wilson et al. (2011), future experiments could use a test battery of other CI parameters (e.g., different carrier rate, asymmetric or ramped pulse shapes, coding intensity via duration instead of amplitude, amplitude modulation) to precisely target and engage more O-units.

Altogether, Johnson et al. (2016) provide solid insight into how the brain responds, or fails to respond, to CI stimulation. The findings will provide crucial guidance for the development of next-generation CIs that target neuronal circuits normally engaged by sound and eventually have potential to improve the outcome of CIs.

## Synthesis

Reviewing Editor: Darcy Kelley, Columbia University Decisions are customarily a result of the Reviewing Editor and the peer reviewers coming together and discussing their recommendations until a consensus is reached. When revisions are invited, a fact-based synthesis statement explaining their decision and outlining what is needed to prepare a revision will be listed below. The following reviewer(s) agreed to reveal their identity: Cory Miller, Andrew Dykstra

Both reviewers were in agreement that the commentary provides a valuable discussion of an important topic. Reviewer one's major comments focus on the feasibility of some of the proposed experiments in marmosets; reviewer two had a number of specific comments on other topics. These comments are as follows:

This manuscript provides a summary review of a recent study of auditory cortical responses following cochlear implantation in marmoset monkeys. The authors accurately describe the work and highlight some of the more notable findings from the study. Although overall the piece is well written, the authors do not seem to titer their hypotheses and future experimental ideas to what is possible in marmosets. Some of the experiments are not feasible in marmosets currently, while it is not clear what a marmoset experiment would look like to address the issues related to experience. Given that it is not yet known, at least to my knowledge, whether the same pattern of responses in AC would occur in rodents after CI implementation, I would strongly encourage the authors to titer their ideas within what is feasible in marmosets. My more specific comments are below.

- The PV optogenetics experiment proposed by the authors would not be possible in marmosets right now. There is currently no promoter available that could bias viral expression to PV interneurons in a primate species. Gord Fishell and colleagues did recently develop a 'mini-promoter' that biases expression to interneurons in vertebrates, including marmosets, but not to any specific class within this cell group. Perhaps this experiment could be performed in a mouse, but differences in the auditory system between mouse and monkey might make interpreting the results challenging. As a result, this experiment, while certainly intriguing to test the authors hypothesis, should be regarded more as a thought experiment than something that could be done at this point.

- The second to last paragraph is difficult to follow. Certainly, the effects of training and experience-dependent plasticity on AC responses in rodents is well documented. But it is not entirely clear how this relates to the Wilson studies or, more importantly, what precisely is the experiment in marmosets. A more careful description of how these results could for the basis for a future marmoset study, along with predictions, would be helpful.

- The following sentence in paragraph 2 is awkward. 'Johnson et al. (2016) uses a new way of thinking about CI design that includes the brain'. I think that I know what you are getting at, namely distinguishing between central and periphery, but discussing it as novel to 'think about the brain' in CI is misleading. I would suggest rewording this sentence and the next one to the peripheral/central mechanistic distinction. Certainly the 'Brain' is on the mind of the CI community.

And also:

This is a well-thought out discussion of an interesting target article, and goes beyond a mere rehashing of the results by providing specific testable hypotheses about how auditory cortex responds to CI stimulation. Below, I offer some suggestions for how the authors might improve the manuscript further.

Specific points:

- At the end of the first paragraph, the authors should mention cochelar synaptopothy, i.e., the relatively recent notion that the primary cause of hearing deficits experienced by older, middle-aged, and even some younger listeners in noisy environments may be a loss of certain auditory nerve fibers rather than hair cells. As cochlear implants work via stimulation of such nerve fibers, this would seem to have important implications for their design.

- Page 2, paragraph 3: “The common marmoset (Callithrix jacchus) is the closest researcher can get to study how the human brain processes auditory information:”

This is misleading. Although it is challenging due to electromagtnetic artifacts caused by the CI, it is indeed possible to study the same issues in humans, via EEG or MEG, for example. The authors should qualify this statement to be more specific about the fact that studying this issue in marmosets allows one to examine single-unit responsiveness, which would indeed be extremely difficult if not impossible in humans (unless there is a recipient of a cochlear implant who also has medically intractable epilepsy; even then, measuring single units from auditory cortex would not necessarily be possible).

- Page 2, paragraph 3: “commutative”

Did the authors mean to write “communicative”?

- Page 2, paragraph 3: “... a neuron in one hemisphere is excited by inputs from both ears ...”

This is oversimplified. There are strong differences between contralateral and ipsilateral stimulation, with contralateral stimulation typically being much more effective. In fact, as I see it, this is one limitation of the target paper, i.e., that it may have been difficult to “functionally localize”, via acoustic stimulation, responsive neurons in the hemisphere contralateral to the implant.

- Page 2, paragraph 4:

“good” → “well”

- Page 2, paragraph 4: “One striking finding of the present work is that CI stimulation was surprisingly inefficient in activating as many neurons as comparable acoustic stimulation.”

It might be useful to elaborate a bit on how the authors of the original study attempted to match acoustic and CI stimulation.

- Page 2, paragraph 5: “CI-stimulation showed mainly to engage neurons with O-shaped FRA's while CI non-responsive cells typically had O-shaped FRA's (Johnson et al., 2016).”

Did the authors mean to write: “CI-stimulation showed mainly to engage neurons with V-shaped FRA's while CI non-responsive cells typically had O-shaped FRA's (Johnson et al., 2016).”

- Page 2, paragraph 6: “The fact that non-responsive cells were mainly located in upper cortical layers and CI-responsive cells in the middle thalamo-recipient layers, suggested that the CI signal is received by cortical structures but is hindered in the further intracortical processing.”

Please remove the comma as wellas the second and fourth instances of “the”.

- Page 3, last paragraph: “The most common type is parvalbumin-positive interneurons (PVs) that target the pyramidal cell bodies and gate feed-forward thalamocortical auditory inputs (Hamilton et al., 2013).”

The authors could maybe be more specific about exactly where the “gate” is, i.e. at the output of the layer III/IV neurons.

- Page 4, first full paragraph: “Therefore, an important caveat of the work by Johnson et al. (2016) is that even though recordings were made up to 14 months after CI implantation, animals only received passive stimulation during the experimental sessions. The results might thus relate to mechanisms in recently implanted CI user before any plasticity takes place, rather than in an experienced CI user.”

It is unclear what the authors mean here. 14 months of even passive stimulation, especially if combined with communication with conspecifics (which I assume took place) would seem sufficiently long for the auditory cortex to have reorganized. Do the authors mean to distinguish between task-relevant and task-irrelevant stimulation and what effect this might have on CI-stimulated units? If so, this needs to be clarified.

- Page 4, second full paragraph:

“off” → “of”

- Page 4, second full paragraph: “Nonetheless, many CI users face challenges with hearing in dynamic and noisy environments, which is partially due to limited dynamic range (5-15 dB compared to 0-120 dB in normal hearing) and a poor ability to discriminate steps in amplitude of a sound (Moore, 2003; Wilson et al., 2011).”

These seem like a huge caveats that maybe warrant a bit more discussion?
